# The Effect of Hypertension and Diabetes Management in Southwest China: A Before- and After-Intervention Study

**DOI:** 10.1371/journal.pone.0091801

**Published:** 2014-03-14

**Authors:** Xiaohua Liang, Jie Chen, Youxue Liu, Chunling He, Tingyu Li

**Affiliations:** 1 Ministry of Education Key Laboratory of Child Development and Disorders, Key Laboratory of Pediatrics in Chongqing, Chongqing International Science and Technology Cooperation Center for Child Development and Disorders, Children's Hospital, Chongqing Medical University, Chongqing, People's Republic of China; 2 Centers for Disease Control and Prevention of Jiulongpo District, Chongqing, People's Republic of China; University of Sao Paulo, Brazil

## Abstract

**Background:**

Non-communicable diseases are leading causes of disease burden in middle income countries. Little evidence exists to determine if the primary healthcare system can effectively manage non-communicable diseases. The purpose of this study was to examine the effectiveness of hypertension and diabetes management by the primary healthcare system.

**Methods:**

We used individual level data from the 2009 National Basic Public Health Services System to assess the effectiveness of hypertension and diabetes interventions on fasting plasma glucose, and blood pressure. We analyzed the associations between fasting plasma glucose, systolic or diastolic blood pressure and risk factors. The estimated average intervention effect on data balanced with confounding variables was assessed.

**Results:**

9543 individuals who had data for fasting plasma glucose, systolic blood pressure and diastolic blood pressure were included in this analysis. This study included 6681 patients with hypertension and 2222 with diabetes. The intervention lowered mean fasting plasma glucose by 0.5 mmol/L (0.4–0.6), lowered mean systolic blood pressure by 3.5 mm Hg (3.2–3.7), and lowered diastolic blood pressure by 2.9 mm Hg (2.7–3.2). Individuals who received medicinal treatment had 1.3 mmHg (0.8 to 1.8, P<0.01) lower diastolic blood pressure and 0.6 mmol/L (0.5–0.8, P<0.01) lower fasting plasma glucose than those who did not receive medicine. Generalized linear model indicated that medicinal treatment and baseline systolic blood pressure were significant positive predictors of change in systolic blood pressure. Age, living in urban areas and diabetic complications were significant negative predictors of change for systolic blood pressure.

**Conclusion:**

The National Basic Public Health Services System in China using trained community healthcare workers and well-established guidelines can be effectively implement non-communicable disease prevention and management care paradigms.

## Introduction

Hypertension and diabetes are primary contributors to the burden of disease in countries at all stages of economic development [1]–[2]. It was estimated that 26.4% of the adult population in 2000 had hypertension, and 29.2% by the year 2025 [3]. The number of adults with hypertension in 2025 was predicted to increase by about 60% to a total of 1.56 billion [3]. Similarly, the worldwide prevalence of diabetes, which was 8.3% in 2011, is predicted to rise to 9.9% by 2030 [4]. Although diabetes has a lower prevalence than hypertension, it has a substantially socioeconomic impact. Globally, 12% of health expenditure was spent on diabetes in 2010 [5]. In China, hypertension and diabetes are both important risk factors for mortality and contribute a lot to the economic burden of preventable disease [6]–[7].

The greatest increases in the prevalence of hypertension and diabetes have occurred in countries of economic transition, including China [8]. This dramatic increase has the potential to put severe strain on healthcare systems in China. Although population growth and aging have led to a rise in the absolute burden of chronic diseases, age-specific mortality, as well as the incidence of cardiovascular diseases and some other non-communicable diseases have decreased in high income countries [9]. This success is partly attributable to a nationwide reduction in major risk factors, including smoking cessation and management of high blood pressure, hypercholesterolemia and hyperglycaemia [6] [10]–[11]. Despite the success at addressing these risk factors in high-income countries, the prevalence of these risk factors has increased or remained unchanged in many low-income and middle-income countries [6] [10]–[11]. Dietary, lifestyle, regulatory, and pharmacological interventions can lower major risk factors for cardiovascular disease, and have contributed to decreased mortality in high-income settings [6] [12]–[13]. However, the ability to identify people at high risk for cardiovascular disease, to deliver interventions, and to ensure compliance to these interventions is constrained by the number and cost of physicians and health facilities.

Interventions initiated in a primary care setting may provide a cost-effective mechanism for the management of cardiovascular risk factors in low-income and middle-income countries [14]. However, the absence of effectiveness research in the primary care setting is a major obstacle to the forming specific policies pertaining to the management of cardiovascular disease and its risks. The purpose of this study was to assess the effectiveness of a community-based intervention conducted by the National Basic Public Health Services. We hypothesized that a hypertension and diabetes management program utilizing general practitioners would lower cardiovascular risk factors.

## Materials and Methods

### Intervention method and data sources

The enrollment and research plans were reviewed and approved by the institutional ethical committee of the Centers for Disease Control and Prevention of Jiulongpo District in Chongqing, China (reference number:066/2008). Informed written consent was obtained from patients by a general practitioner(GP). As defined by this study, a GP is a medical practitioner who treats acute and chronic illnesses and provides preventive care and health education to patients. These practitioners are different from the specialists who treat a specific condition or specific body system. The National Basic Public Health Services system used GPs to offer primary health care in the community for 11 projects, including health profiling, hypertension management, planned immunity for children, diabetes management, psychopath management, elderly care, child care, maternal care, health education, sanitation management, and infectious diseases and public health emergencies. In 2009, several provinces utilized the services of these GPs to start a community management program specific to hypertension and diabetes. In this study, we will analyze the hypertension and diabetes intervention conducted as part of National Basic Public Health Services.

GPs were trained to identify high-risk groups. The high-risks groups were defined as 1) individuals aged 18 years or older who had a family history of hypertension or diabetes, or 2) overweight individuals diagnosed with hypertension and diabetes via a community health screening. High-risk individuals were referred to physicians who visited the community health service centre for blood pressure and hyperglycemia testing and they got treatment/health management if they were high risk. Diagnosis, treatment, and lifestyle advice were provided and details were recorded by the GPs. The dates of subsequent visits to the GPs to address hypertension and diabetes issues were also noted.

Patients diagnosed with diabetes or hypertension participated in a single physical examination with at least four follow-up visits from a medical team that consisted of the GPs, nurse, clinical pathologist and public health doctor. These visits were carried out on an annual basis at the community health care. In addition to the initial screening, the GPs were responsible for training sessions on healthy diet and lifestyle modifications. The nurses provided the physical examination, including blood pressure and body mass measurements for those with hypertension and diabetes. The clinical pathologist also performed a pathological examination and the public health doctor offered health education and directed patients to public health events. To ensure the quality and adherence to follow-up care, the GPs group made household visit by bicycle in rural areas that were not accessible to automobiles.

We used data from the 2009 National Basic Public Health Services System (NBPHSS) to measure the coverage of treatment for hypertension and diabetes, and to estimate the effectiveness of the intervention at reducing blood pressure (BP) and fasting plasma glucose (FPG) after one year of management. We included 6,681 individuals aged 20 years or older with hypertension and 2,222 participants with diabetes aged 20 years or older who attended the laboratory sessions. We used clustered sampling to create a representative sample of the rural and urban populations of Chongqing from NBPHSS. 1,513 patients who were diagnosed with both hypertension and diabetes in this study were included in this study. The NBPHSS is a web database that includes a questionnaire with name, sex, age, ID, community of residence, systolic and diastolic blood pressure, and FPG. Participants were instructed to fast for at least 8 hours prior to FPG. The questionnaire also included questions about drug use for hypertension or diabetes treatment. Drug use was confirmed through a review of medical records or evaluation of the patient's medicine box at the follow-up or physical examination.

The diagnosing of hypertension and diabetes started in 2009, after diagnosed they were managed by GP and have not finished until now. Respondents were classified as having diabetes if 1) their FPG concentration was 7 mmol/L or higher, 2) they reported having been diagnosed with diabetes, or 3) they were taking glucose-lowering drugs [15]. In the absence of symptomatic hyperglycemia with a single laboratory test that was in the diabetes range, a repeat confirmatory FPG laboratory test was done within one week. If both laboratory results were above the diagnostic cut points, the diagnosis of diabetes was confirmed. According to the guidelines from the seventh report of the Joint National Committee on Prevention, Detection, Evaluation, and Treatment of High Blood Pressure (JNC 7), respondents were classified as having hypertension if 1) their SBP was equal or greater than 140 mmHg, 2) their DBP was equal or greater than 90 mmHg, 3) they reported having been diagnosed with hypertension, or 4) they were taking drugs to lower their blood pressure [16]. In order to ensure accuracy, manually blood pressure was measured three times and followed up by the validated Omron Hem-7071-CP (Omron Healthcare, Kyoto, Japan) upper arm automated monitor.

**Figure 1:**
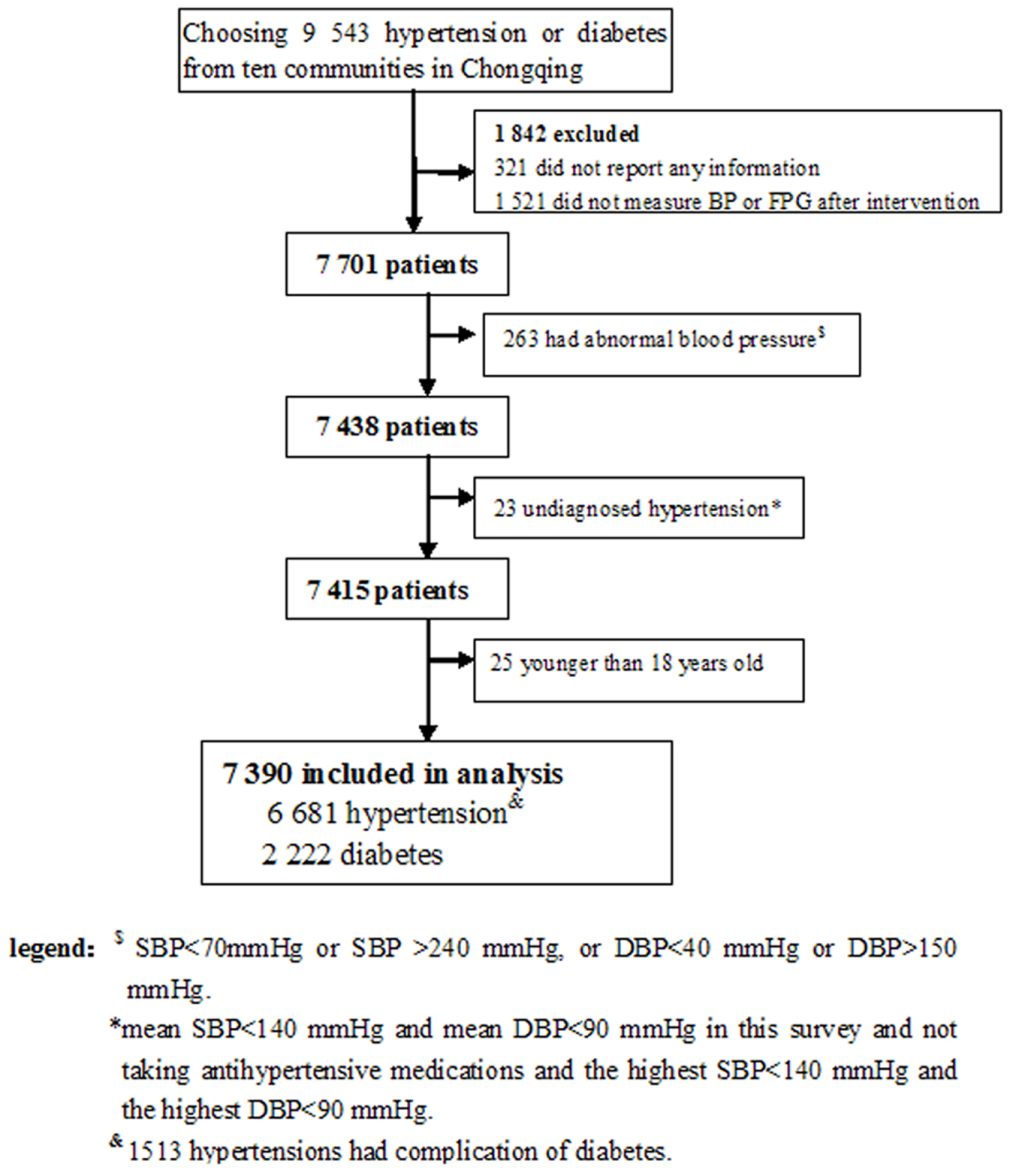
Flow of Patients from Recruitment to Finally been Included in the Assessment.

According to previous described eligibility criteria, the study included patients diagnosed with hypertension and diabetes. The study was designed to enroll 9,543 patients, and 7,390 patients were used for analysis (Figure 1). The study included 6,681 patients with hypertension and 2,222 patients with diabetes, 1513 patients were diagnosed with both hypertension and diabetes. The sample size in the hypertension management group was powered to detect clinically meaningful differences in both SBP and DBP of 2 mmHg after one year intervention. The sample size provided 95% power to detect differences in both SBP and DBP, and assumed a normal approximation to compare 2 independent means. The assumed standard deviations for SBP and DBP were both 10 mmHg and 10 mmHg. Sample size was estimated using a 2-sided α of 0.01 and a 70% response rate. The sample size in the diabetes management group provided 95% power to detect clinically meaningful differences in FPG, which was defined as 0.5 mmol/L, using a paired pre-post design. We assumed that the difference in the response is normally distributed with a standard deviation of 2, a 2-sided α of 0.01 and a 95% response rate.

### Statistical Analysis

Descriptive statistical analysis was used to analyze the demographic characteristics of the sample. We estimated the average effect of treatment on SBP and DBP for individuals with hypertension, and we also estimated the average effect of management on FPG for individuals with diabetes using the paired Student's t tests [17]. We adjusted for age, sex, medicine treatment, rural residency, complications and baseline FPG, SBP and DBP to estimate the average effect of treatment on FPG, SBP and DBP. A generalized linear model (GLM) [18] was used to account for unmeasured factors that might affect the value of SBP, DBP or FPG. In this model the decrease in SBP, DBP or FPG was used as the dependent variable and medication treatment, sex, age, rural residency and complications of hypertension or diabetes were used as the independent variable. We did the above analyses on all individuals with SBP, DBP and FPG data in the NBPHSS. All analyses were done separately for diabetes and hypertension. The analyses were performed using SAS Software (version 9.13). Tests of significance were two-tailed and P<0.05 was considered statistically significant.

### Patient Characteristics

Demographic characteristics of patients with hypertension and diabetes are shown in Table 1. In total, 6,681 patients with hypertension (2,824 men; mean age 60.2±10.9 years) and 2,222 patients with diabetes (920 men; mean age 59.8±10.8 years) were included in this analysis. Of these individuals, 2,645 patients with hypertension and 1,310 patients with diabetes lived in urban areas. 4,036 patients with hypertension and 912 with diabetes lived in rural areas. 2,428 (36.3%) patients with hypertension and 884 (39.8%) patients with diabetes received medication treatment for their respective conditions. Hypertension and diabetes often occurred in combination. The rate of combined disease was 22.65% (1,513/6,681) for patients with hypertension, and 68.09% (1513/2222) for patients with diabetes.

**Table 1:** Demographic Characteristics of the Sample.

Characteristics	Hypertension		Diabetes	
	n	(%)	n	(%)
Sample size	6681	100	2222	100
**Age group**				
18–39 years	454	6.8	152	6.8
40–49 years	1263	18.9	439	19.8
50–59 years	2259	33.8	772	34.7
60–69 years	2074	31.0	650	29.3
≥70 years	631	9.5	209	9.4
**Sex**				
Male	2824	42.3	920	41.4
Female	3857	57.7	1302	58.6
**Community**				
Urban	2645	39.6	1310	59.0
Rural	4036	60.4	912	41.0
Treatment*				
No	4253	63.7	1338	60.2
Yes	2428	36.3	884	39.8
**Combination disease** &				
No	5168	77.35	709	31.91
Yes	1513	22.65	1513	68.09

Notes:*Prescription medication treatment for hypertension or diabetes

& Hypertension and diabetes present in the same individual.

## Results

### Analyses of effect size

The effect of hypertension and diabetes management on SBP, DBP and FPG of individuals was analyzed as a whole (Table 2) as well as subgroups based on area of residence, age, sex and use of medicine. Patients who participated in hypertension health management by a GP group demonstrated a mean reduction in SBP of 3.5 mm Hg [95% CI: 3.2–3.7 mm Hg] over a median follow-up of one year. In the subgroup analysis, there was a significantly greater reduction in SBP by those who live in rural areas compared to urban areas (mean difference −0.5 mmHg, 95% CI: −1.0–0.1 mmHg, P = 0.08) and for those between the ages of 40 and 50 compared to other age groups. There was no significant effect of sex and use of medicine treatment on the intervention outcome in SBP. The pooled difference in DBP change was 2.9 mmHg (95% CI: 2.7–3.2 mmHg, P<0.01), indicating that the reduction of DBP value was 2.9 mm Hg lower after intervention compared with before intervention. Subgroup analysis revealed that female individuals showed significantly greater reduction in DBP than did male individuals (3.2 mmHg vs. 2.7 mmHg, P = 0.03). There was a significant effect of residence area and those who lived in urban areas than had significantly greater reduction in DBP that those in rural areas. Similarly, subjects who used medication had a greater reduction in DBP than those without medication treatment (Table 2). There was no effect of age in the subgroup analysis in DBP.

**Table 2:** The effect of diabetes and hypertension management on FPG, SBP and DBP.

Variable	Systolic blood pressure (n = 6681)		Diastolic blood pressure (n = 6681)		Fasting plasma glucose (n = 2222)	
	Mean (95% CI)	P value	Mean (95% CI)	P value	Mean (95% CI)	P value
**Sample size**	6681		6681		2222	
Total	3.5 (3.2–3.7)	<0.01	2.9 (2.7–3.2)	<0.01	0.5 (0.4–0.6)	<0.01
**Sex**						
male	3.4 (3.1–3.8)	<0.01	3.2 (2.8–3.5)	<0.01	0.5 (0.4–0.6)	<0.01
female	3.5 (3.1–3.8)	<0.01	2.7 (2.4–3.0)	<0.01	0.6 (0.5–0.7)	<0.01
differ	0.1 (−0.5–0.5)	0.92	0.5 (0–1.0)	0.03	−0.1 (−0.3–0.1)	0.21
**Community**						
urban	3.2 (2.8–3.6)	<0.01	3.7 (3.4–4.1)	<0.01	0.7 (0.6–0.8)	<0.01
rural	3.6 (3.3–4.0)	<0.01	2.3 (2.0–2.6)	<0.01	0.2 (0.2–0.3)	<0.01
differ	−0.4 (−1.0–0.1)	0.08	1.4 (1.0–1.9)	<0.01	0.5 (0.3–0.7)	<0.01
**Age group**						
18–39 years	3.1 (2.3–3.9)	<0.01	3.2 (2.4–3.9)	<0.01	0.7 (0.3–1.1)	<0.01
40–49 years	4.7 (4.1–5.2)*	<0.01	3.2 (2.7–3.7)	<0.01	0.7 (0.5–0.9)	<0.01
50–59 years	3.6 (3.2–4.1)	<0.01	3.2 (2.8–3.6)	<0.01	0.5 (0.4–0.6)	<0.01
60–69 years	2.8 (2.4–3.3)	<0.01	2.3 (1.8–2.7)	<0.01	0.4 (0.3–0.6)	<0.01
≥70 years	2.8 (2.0–3.7)	<0.01	2.9 (2.1–3.6)	<0.01	0.5 (0.3–0.7)	<0.01
differ		<0.01		0.01		0.17
**Treatment**						
No	3.4 (3.1–3.7)	<0.01	3.3 (3.0–3.6)	<0.01	0.3 (0.2–0.3)	<0.01
Yes	3.5 (3.1–3.9)	<0.01	2.1 (1.7–2.4)	<0.01	0.9 (0.7–1.0)	<0.01
differ	−0.1 (−0.6–0.4)	0.69	1.2(0.8–1.8)	<0.01	−0.6 (−0.8–−0.5)	<0.01

*:Means are significantly different from other group.

The pooled difference in FPG change was 0.5 mmol/L (95% CI: 0.4–0.6), indicating that on average FPG value was reduced 0.5 mmol/L after the intervention. There was a significant effect of area of residence, and individuals in urban areas had a mean reduction of 0.7 mmol/L (95% CI: 0.6–0.8) compared to 0.2 mmol/L (95% CI: 0.2–0.3) in rural areas (mean difference 0.5 mmol/L, 95% CI: 0.3–0.7 mmol/L, P<0.01). There was no significant effect of age group on FPG values with differences 0.3 mmol /L or less (P = 0.17). Those who used medication had a mean change of 0.9 mmol/L (95% CI: 0.7–1.0) compared to 0.3 mmol/L (95% CI: 0.2–0.3 mmol/L) in those who did not use medication (mean difference 0.6 mmol/L, 95% CI: 0.5–0.8 mmol/L, P<0.01).

### Average effect adjusted covariates

**Figure 2:**
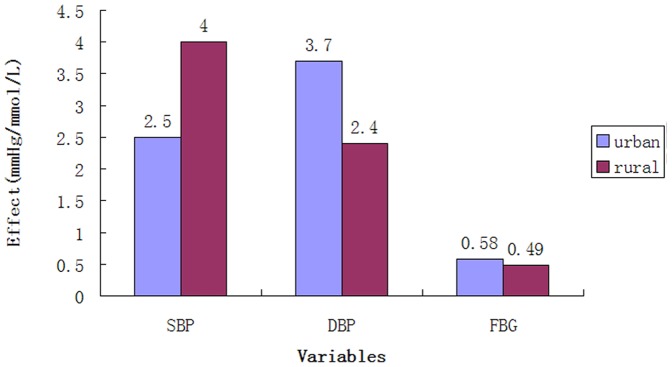
Effectiveness after adjusted covariates.

Hypertension health management reduced SBP and DBP by a mean of 2.5 mmHg (standard error = 0.19) and 3.7 mmHg (standard error = 0.16) in urban areas and 4.0 mmHg (standard error = 0.15) and 2.4 mmHg (standard error = 0.12) in rural areas after adjusting for age, sex, medicine treatment, diabetic complications and baseline blood pressure (Figure 2). FPG decreased 0.58 mmol/L (standard error = 0.04) and 0.49 mmol/L (standard error = 0.05) in urban and rural areas, respectively, after adjusted for age, sex, medicine treatment, hypertension complications and baseline FPG.

The GLM analysis revealed that age, urban areas, and diabetic combinations were strongly associated with an increase in SBP (P<0.01), while medication treatment and baseline SBP were associated with a decrease in SBP (P<0.01) (Table 3). The association between sex and SBP was not statistically significant (P = 0.62). Age, residence in urban areas, diabetic combinations and baseline DBP were statistically associated with the reduction of DBP, while medicinal treatment was significantly associated with the increased DBP (P<0.01). Baseline blood pressure (P<0.01) and baseline FPG (P<0.01) were significantly related to improvements in FPG, while age, sex, area of residence, and medicinal treatment were not statistically related to change in FPG.

**Table 3:** The results of the GLM regression for blood pressure and fasting plasma glucose.

Parameter	Systolic blood pressure (n = 6681)		Diastolic blood pressure (n = 6681)		Fasting plasma glucose (n = 2222)	
	Estimate (SE)	P value	Estimate (SE)	P value	Estimate (SE)	P value
Intercept	−41.47 (1.28)	<.01	−53.15 (1.04)	<.01	−2.18 (0.2)	<.0001
**Sex**						
Male	−0.11 (0.23)	0.62	0.02 (0.19)	0.9337	0.05 (0.06)	0.42
Female (ref.)	0	.	0	.	0	.
Age (continuous)	−0.02 (0.01)	0.04	0.05 (0.01)	<.01	−0.01 (0)	0.06
**Community**						
Urban	−1.5 (0.26)	<.01	1.28 (0.21)	<.01	0.08 (0.07)	0.26
Rural (ref.)	0	.	0	.	0	.
Medicinal treatment	1.53 (0.25)	<.01	−0.96 (0.21)	<.01	0.07 (0.07)	0.35
** Complication**						
Diabetes	−0.41 (0.14)	<.01	0.25 (0.11)	0.03	—	—
Hypertension	—	—	—	—	−0.28 (0.06)	<.01
**Baseline SBP**	0.33 (0.01)	<.01	—	—	—	—
**Baseline DBP**	—	—	0.61 (0.01)	<.01	—	—
**Baseline FPG**	—	—	—	—	0.42 (0.01)	<.01

## Discussion

In our representative analysis, management of hypertension and diabetes by the community health service centers in the Southwest of China was effective. There was a greater improvement in SBP in rural areas compared to urban areas, although the effect decreased with the increase in age. There was a significant difference in the treatment effect for males and females for DBP, with males having a slightly more favorable outcome. Younger age was also associated with lower DBP one year after the intervention, as was the case with SBP.

The positive outcomes from this intervention are likely due to the role of the GP in the healthcare. The GPs are responsible for individual-based prevention and control services, including diet and lifestyle education and monitoring treatment adherence on a regular basis. This direct oversight from the GP promotes positive behavior changes and compliance with medicine treatment. Under the NBPHSS program, physicians provide patients with specific treatment guidelines and health management care.

We believe that the quarterly visits to monitor patient medication adherence and provide medical care if symptoms of hyper-glycaemia or hyper-blood develop, are just as important as the initial diagnosis. Because there is little direct medical contact and management for chronic diseases in rural areas, compliance with medication and physician visits is often the responsibility of the patients. The absence of rigorous follow-up exams in rural areas may partially explain the relationship between rural residence and reduced effectiveness at lowering FPG.

Our results are in accordance with outcomes from other trials from other geographical regions [19]–[21] that have found that health management is associated with improved outcomes, including better diet quality, increased physical activity and decreased risk of cardiovascular disease [22]–[27]. To our knowledge, no previous longitudinal study analyzed the effects of health management on SBP, DBP and FPG in Southwest of China. This study utilized a large representative health examination cohort survey with high quality, measured data to detect individual-level risk factors and socio-demographic variables associated with cardiovascular disease. However, this study was limited to the data provided by NBPHSS and did not include a comparative control group in the analysis. In addition, repeated surveys would have allowed examination of not only the cross-sectional association, but also the effect of change in SBP, DBP and FPG. Follow-up studies of individual patients with measurements before and after management would increase our confidence in the intervention effects. However, because our findings for the effect of health management on glucose concentrations and blood pressure were statistically significant, residual confounding by other common risk factors is less probable.

In this study, we applied GLM statistical approaches: 1) to analyze the main risk factors that impacted the changes in blood pressure and FPG; 2) to control for potential confounding effects of the full NBPHSS sample; 3) to assess the average treatment effect of data balanced with potential confounding. Our conclusions about intervention effectiveness were consistent between the two analysis methods, which increased our confidence in these findings. We were unable to analyze the effects of the quality of GP's patient management or the role of healthcare and transportation infrastructures on the effectiveness of risk-factor management. We were also not able to measure health management costs in this study because such data were not available.

The number of GPs in Southwest China was originally determined when the program initiated in 2009. In the past several years, the role of the GP has been greatly expanded, while the number of practitioners has not changed. As hypertension and diabetes programs continue to grow and the responsibility of each GP increases, the amount of time each GP can spend on an individual patient is reduced. While the nationwide economic situation impacts the government's investment in NBPHS, it is important to consider the regional differences and needs when assessing this investment. Southwest China is far less developed than other regions of China and the government investment in NBPHS must be evaluated on a local level. Comparisons with respect to program effectiveness and per-capita GP need cannot be made with more developed areas in China. The current financial situation has rendered the primary health-care system in Southwest China virtually ineffective at making strides to continue to decrease blood pressure and FPG on a region-wide level. It is recommended that investments in this region shall be made to further these programs, to address the leading metabolic risk factors for mortality in China.

Therefore, the NBPHSS has several challenges. The system needs to increase its scope so that it can have a more prominent role in hypertension and diabetes diagnosis. This includes addressing non-communicable diseases and risk factors, such as coronary heart disease and high cholesterol. A more fundamental reform should shift the focus of care towards the management of multi pre-risk factors [13]. Given the beneficial role of GPs in this study, increasing the number of GPs in areas where the number of workers is low and the number of patients is high would strengthen the impact of this care paradigm. Randomized studies have shown that diet, lifestyle, and clinical interventions can slow or even reverse hypertension and hyperglycemia under the condition of good compliance [28]–[33]. This is consistent with our findings, which support the feasibility and need of the integrating the management of non-communicable diseases into the primary health-care system.

## Conclusions

This research showed that community management of hypertension and diabetes improved BP and FPG metrics. Despite the relatively large sample size, this pre- post analysis of a community-based intervention had some limitations. Further well-designed randomized controlled trials with large sample sizes are needed to further demonstrate how community intervention can improve blood pressure, FPG, blood lipid control. The cost-effectiveness of such programs should also be explored.
